# Markedly lower follow-up rate after liver biopsy in patients with non-alcoholic fatty liver diseases than those with viral hepatitis in Japan

**DOI:** 10.1186/1756-0500-4-341

**Published:** 2011-09-09

**Authors:** Hidenori Toyoda, Takashi Kumada, Seiki Kiriyama, Makoto Tanikawa, Yasuhiro Hisanaga, Akira Kanamori, Toshifumi Tada

**Affiliations:** 1 Department of Gastroenterology, Ogaki Municipal Hospital, Ogaki, Japan

**Keywords:** Non-alcoholic fatty liver disease, non-alcoholic steatohepatitis, simple steatosis, follow-up, compliance

## Abstract

**Background:**

Patients with non-alcoholic fatty liver diseases (NAFLD) are recommended to have periodic follow-up exams because these patients are at increased risk of the presence of non-alcoholic steatohepatitis (NASH), which can lead to cirrhosis or hepatocellular carcinoma. We investigated the follow-up status of NAFLD patients after a liver biopsy examination.

**Methods:**

We compared the follow-up rates of NAFLD patients who had received an ultrasonography-guided liver biopsy and patients who had received a liver biopsy for chronic viral hepatitis (hepatitis B or C).

**Results:**

The 1- and 3-year follow-up rates after the liver biopsy were 92.7% and 88.3% for patients with chronic HBV infection, and 93.4% and 88.2% for patients with chronic HCV infection, respectively. In contrast, the follow-up rates for NAFLD patients were 77.6% and 49.9%, respectively, which were significantly lower than those of patients with chronic viral hepatitis (*p *< 0.0001). Among NAFLD patients, the respective 1- and 3-year follow-up rates were 73.0% and 44.6% for patients with simple steatosis and 80.0% and 52.4% for patients with NASH based on a pathologic diagnosis, without significant difference between these two subgroups (*p *= 0.5202).

**Conclusions:**

The outpatient-based follow-up rate after a liver biopsy was significantly lower in NAFLD patients compared to patients with chronic viral hepatitis, regardless of the presence of NASH. It is important to determine how to maintain regular hospital visits for NAFLD patients, preventing patient attrition.

## Background

Nonalcoholic fatty liver disease (NAFLD) encompasses a histological spectrum that ranges from simple steatosis to nonalcoholic steatohepatitis (NASH), which can progress to cirrhosis and end-stage liver disease. NAFLD is one of the most common liver diseases in both Western and Asian countries [1-5], affecting 30% of the general Western adult population [6].

Because NAFLD includes patients with NASH that can progress to cirrhosis [1,7,8] and because it is still controversial whether simple steatosis converts to NASH, it is important to carefully monitor NAFLD patients at regular follow-up exams. Furthermore, in the absence of treatment modalities with proven efficacy, outpatient-based weight management is currently an important treatment for NAFLD patients. However, it is unclear whether NAFLD patients have a sufficient understanding of the importance of periodic follow-up visits compared to patients with viral hepatitis or other liver diseases. Whereas the follow-up rate of patients with chronic viral hepatitis (chronic hepatitis B or C) is high in Japan, the follow-up rate of patients with NAFLD, in comparison to patients with viral hepatitis, is not clarified.

In the present study, we investigated the follow-up status of NAFLD patients, including patients with both simple steatosis and NASH, after the disease diagnosis was histologically confirmed by a liver biopsy.

## Methods

### Patients and Follow-up Visits

A total of 610 patients had undergone an ultrasonography-guided liver biopsy examination at our institution to confirm a diagnosis of liver diseases during a 5-year period between April 2003 and March 2008. These patients received a liver biopsy to evaluate of the grade of liver damage (activity of hepatitis), examine the degree of liver fibrosis, and investigate or confirm the diagnosis. The patients included 305 males and 305 females with a mean age of 53.9 ± 12.7 years. All patients underwent an ultrasonography-guided fine needle liver biopsy using a 17 G biopsy needle during their 3 to 4 days of hospitalization. Among these patients, 69 patients had chronic hepatitis B virus (HBV) infection, 354 patients had chronic hepatitis C virus (HCV) infection, and 135 patients had NAFLD by clinical evaluation. We analyzed the follow-up rates of these patient groups after liver biopsy.

The patients were informed of the pathologic results of the biopsy when they visited the outpatient clinic 2 or 3 weeks after the biopsy. Patients with chronic viral hepatitis (hepatitis B or C) were advised to receive regular periodic follow-up exams every 3 to 6 months to monitor liver damage, fibrosis, and the development of HCC. Patients who were diagnosed with NASH were informed that they could potentially progress to cirrhosis and were at increased risk of developing hepatocellular carcinoma (HCC). They were also advised to have regular follow-up visits at our institution every 3 to 6 months. Patients who were diagnosed with simple steatosis were informed that it has not been fully determined if simple steatosis is converts to NASH and were advised to have regular follow-up exams at our institution every 6 months.

Our institution provides appointment-based outpatient care, and most patients who visit the clinic reserve the next available appointment. If a patient did not visit the clinic despite a scheduled appointment, the patient was telephoned, advised to maintain their regular visits, and given a new appointment. This telephone contact was repeated at least three times. Patients were routinely monitored from the biopsy date until the end of December 2010.

The study protocol was in compliance with the Helsinki Declaration and was approved by the institutional review board of the Ogaki Municipal Hospital for the review of patient clinical records. Written informed consent was obtained from patients who continued to be followed up.

### Clinical and Pathologic Diagnosis of Non-alcoholic Fatty Liver Diseases, Simple Steatosis, and Non-alcoholic Steatohepatitis

A patient was clinically diagnosed with a fatty liver before receiving a biopsy if they met made when the following criteria: 1) persistently abnormal liver function tests for more than 3 months, 2) ultrasonographic images showing steatosis, 3) no evidence of alcohol abuse, and 4) exclusion of other liver diseases and other known causes of steatosis based on the results of specific clinical, biochemical, or imaging studies. The ultrasonographic findings of steatosis were based on known criteria that are used to diagnose a fatty liver by ultrasonography (hepatorenal echo contrast, liver brightness, deep attenuation, and vascular blurring). The first two factors were used as definitive criteria, while the last two factors were taken into account as needed. NAFLD was pathologically diagnosed based on pathologic findings in the biopsied liver specimens. The liver biopsy specimens were stained with hematoxylin/eosin, Masson's trichrome, and PAS and then examined by experienced pathologists. A liver with steatosis involving at least 10% of the hepatocytes was considered to have NAFLD. The presence of NASH was defined according to the NAFLD activity score (NAS) as proposed by the Center for Neuroscience Research and National Institutes of Health [9].

### Statistical Analyses

Quantitative values are reported as the mean ± SD. Between-group differences were analyzed by the chi-square test. Differences in quantitative values between two groups were analyzed by the Mann-Whitney *U *test. Follow-up rates were analyzed using the Kaplan-Meier method, and the log-rank test was used to compare the follow-up rates. The date of liver biopsy was defined as time zero when calculating the patient follow-up rates. Patients who continued their follow-up schedule were censored. Patients who missed a follow-up visit were not censored. All *p*-values were two-tailed, and *p *< 0.05 was considered statistically significant.

## Results

### Patient Characteristics

Table 1 shows the clinical characteristics of the patients who underwent a liver biopsy. Patients with chronic HCV infection were significantly older than patients with chronic HBV infection or NAFLD patients (both, *p *< 0.0001). The prevalence of females was significantly higher among patients with chronic HCV infection compared to NAFLD patients (*p *= 0.0094). The serum ALT activity was significantly higher in NAFLD patients than in patients chronically infected with HCV (*p *< 0.0001). In contrast, there were no differences in the serum ALT activity between patients with NAFLD and those with chronic HBV infection (*p *= 0.4773).

**Table 1:** Clinical characteristics of patients who received a liver biopsy.

	NAFLD (n = 135)	Chronic HBV infection (n = 69)	Chronic HCV infection (n = 354)
Age (years: mean ± S.D.)	48.8 ± 14.7	47.1 ± 12.3	56.5 ± 11.0
Sex (male/female) (%)	84(62.2)/51(37.8)	40(58.0)/29(42.0)	172(48.6)/182(51.4)
AST (IU/L: mean ± S.D.)	58.0 ± 42.6	78.2 ± 67.4	55.9 ± 45.4
ALT (IU/L: mean ± S.D.)	94.7 ± 67.8	126.3 ± 119.6	70.9 ± 74.1

NAFLD, non-alcoholic fatty liver disease; HBV, hepatitis B virus; HCV, hepatitis C virus; AST, aspartate aminotransferase; ALT, alanine aminotransferase

### Follow-up Status after the Liver Biopsy

**Figure 1:**
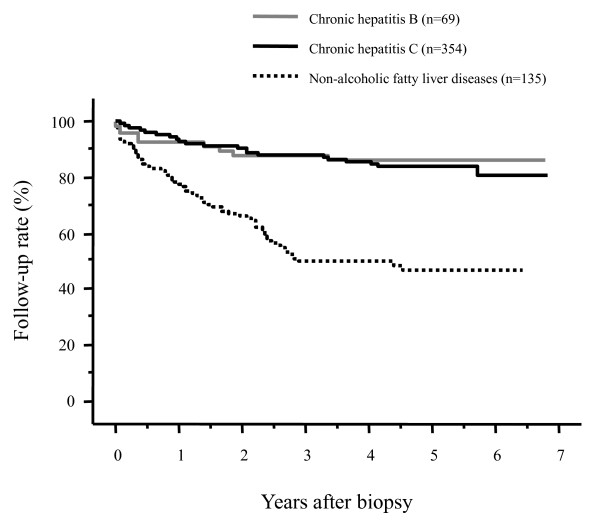
**Post-liver biopsy follow-up rates of patients according to the underlying liver disease**. The follow-up rate was significantly lower in patients with nonalcoholic fatty liver disease than in patients with chronic hepatitis B or C virus infection (both, *p *< 0.0001). There was no difference in the follow-up rate between patients with chronic HBV infection and those with chronic HCV infection (*p *= 0.7299).

**Figure 2:**
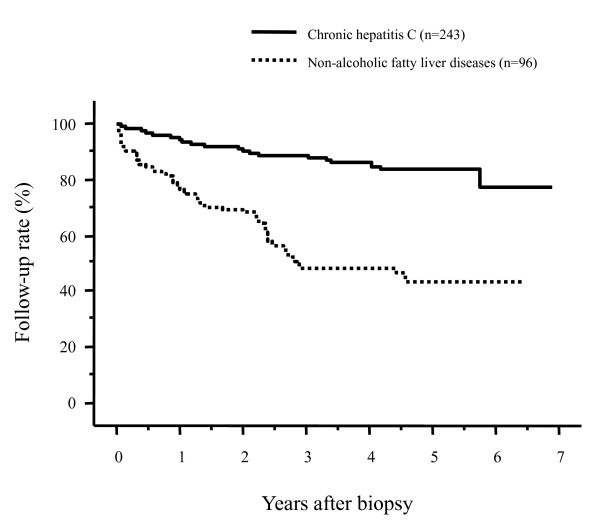
**Comparison of the post-biopsy follow-up rates of patients with chronic hepatitis C virus (HCV) infection and those with nonalcoholic fatty liver disease (NAFLD), focusing on patients who had not been administered medication**. The follow-up rate remained significantly lower in patients with NAFLD than in patients with chronic HCV infection (*p *< 0.0001).

**Figure 3:**
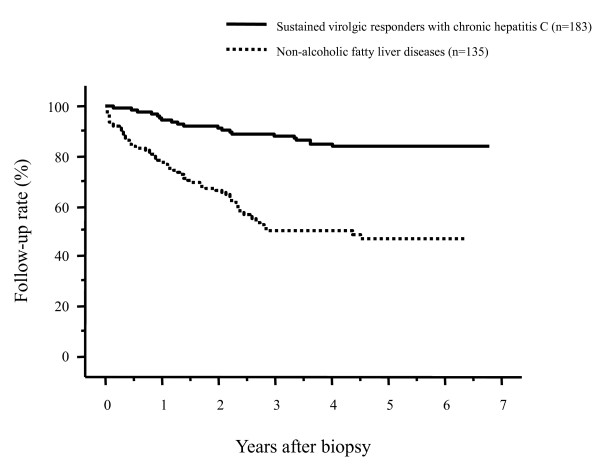
**Comparison of the post-liver biopsy follow-up rates in patients with nonalcoholic fatty liver disease (NAFLD) and patients who had been successfully treated for hepatitis C virus (HCV) with antiviral therapy (sustained virologic responders)**. The follow-up rate was significantly lower in NAFLD patients than in sustained virologic responders for HCV (*p *< 0.0001).

The follow-up rates of patients after the liver biopsy are shown in Figure 1 according to the underlying liver diseases. The 1- and 3-year follow-up rates after liver biopsy were 92.7% and 88.3% for patients with chronic HBV infection, 93.4% and 88.2% for patients with chronic HCV infection, and 77.6% and 49.9% for NAFLD patients, respectively. There were no differences in the follow-up rates between patients with chronic HBV infection and those with chronic HCV infection (*p *= 0.7299). By contrast, the post-liver biopsy follow-up rate was significantly lower in NAFLD patients compared to those with HBV infection or HCV infection (both, *p *< 0.0001); more than 50% of patients discontinued their follow-up exams within 3 years after the liver biopsy. When we compared the post-liver biopsy follow-up rates between patients with chronic HCV infection and those with NAFLD focusing on patients who had not been administered medication, the follow-up rate of patients with NAFLD was significantly lower than that of patients with HCV (*p *< 0.0001, Figure 2). In addition, when we compared the follow-up rates between NAFLD patients and patients who had been chronically infected with HCV but were successfully treated with an antiviral therapy with interferon/peginterferon with or without ribavirin (sustained virologic responders), NAFLD patients had a significantly lower follow-up rate (*p *< 0.0001, Figure 3).

### Comparison of Characteristics Between Non-alcoholic Fatty Liver Disease Patients Who Continued and Discontinued Their Follow-up Exams

**Figure 4:**
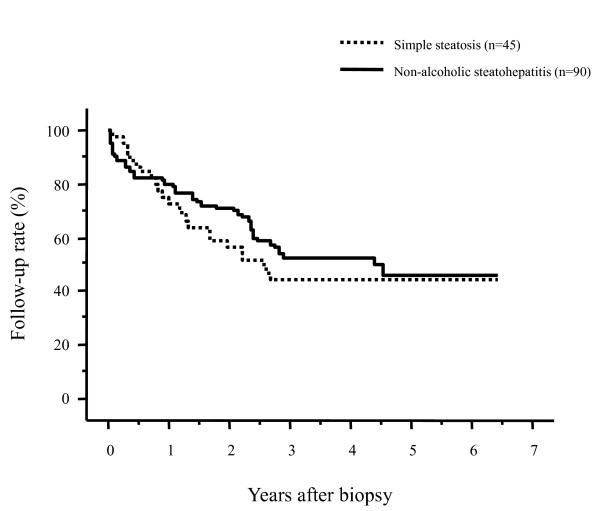
**Comparison of the post-biopsy follow-up rates of patients with nonalcoholic fatty liver disease between patients with nonalcoholic steatohepatitis and those with simple steatosis**. There were no differences in the follow-up rates (p = 0.5202).

When the background characteristics were compared between NAFLD patients who continued and discontinued their periodic follow-up visit (Table 2), the patients who discontinued their follow-up visit were significantly younger than those who continued their regular follow-up exams. The prevalence of NASH and simple steatosis by pathologic evaluation did not differ between patients who had continued and discontinued their follow-up exams. Also, the percentage of patients who had been administered medication did not differ between patients who continued and discontinued their follow-up visit. Figure 4 compares the follow-up rates after the liver biopsy between patients with NASH and those with simple steatosis. The 1- and 3-year follow-up rates were 73.0% and 44.6% for patients with simple steatosis and 80.0% and 52.4% for patients with NASH, respectively, without significant differences between these two groups (p = 0.5202). The presence of NASH, as determined by a pathologic diagnosis, did not influence the follow-up rate after the liver biopsy.

**Table 2:** Comparison of the characteristics of non-alcoholic fatty liver disease patients who continued and discontinued their follow-up exams after a liver biopsy.

	Patients who maintained their follow-up exams (n = 68)	Patients who discontinued their follow-up exams (n = 67)	*p*-value
Age (years: mean ± S.D.)	52.6 ± 13.4	44.9 ± 15.2	0.0035
Sex (male/female) (%)	42 (61.8)/26 (38.2)	42 (62.7)/25 (37.3)	0.9120
AST (IU/L: mean ± S.D.)	59.6 ± 35.0	56.4 ± 49.4	0.1208
ALT (IU/L: mean ± S.D.)	93.6 ± 70.5	95.9 ± 65.4	0.5928
Medication (yes/no) (%)	21 (30.9)/47 (69.4)	18 (26.9)/49 (73.1)	0.7442
Pathologic diagnosis (NASH/simple steatosis) (%)	47 (69.1)/21 (30.9)	43 (64.2)/24 (35.8)	0.6691

AST, aspartate aminotransferase; ALT, alanine aminotransferase; NASH: non-alcohol steatohepatitis.

Among 65 patients who discontinued their follow-up visit for NAFLD, 13 patients (20.0%) had received periodic follow-up examinations for diabetes at the diabetes department of our institution, and 15 patients (23.1%) had received follow-up exams for diabetes by their family physician. However, in both cases, the patients were not examined for liver disease.

## Discussion

Regular outpatient follow-up visits are important for patients with chronic liver diseases. In particular, previous studies have shown that periodic HCC surveillance in patients with liver diseases improves the patient survival due to the detection of early-stage HCC [10,11]. In addition, it is important for NAFLD patients to receive regular advice and be monitored for weight loss, exercise, and specific diets at outpatient clinics to prevent the conversion from simple steatosis to NASH and the progression of NASH. However, there is no study that investigated the outpatient follow-up of patients with NAFLD.

The results of the present study revealed that NAFLD patients have a markedly reduced follow-up rate after they receive their initial diagnostic liver biopsy, in comparison to those with viral hepatitis. The follow-up rate of NAFLD patients was significantly lower than that of patients with chronic HCV infection, even when focusing on patients who had not been administered medication. In addition, the post-biopsy follow-up rate of NAFLD patients was lower in comparison to patients whose chronic HCV infection in was eradicated with antiviral therapy, in whom the follow-up rate was lower compared to patients with ongoing HCV infection [12]. In a previous study on patients with alcoholic liver disease, Trabut et al. reported that a liver biopsy did not further motivate patients to abstain from alcohol [13]. Similarly, in these findings, a liver biopsy did not motivate patients to attend follow-up visits that were designed to control and monitor NAFLD.

When we compared NAFLD patients who continued and discontinued their follow-up visits, there was a significant difference in age between these two subgroups, and younger patients were more likely to miss subsequent follow-up exams. This could be partially due to the difficulty in visiting the hospital due to the limited appointment times, and partly because younger patients tend to be less interested in their health than older patients. Interestingly, there were no differences in the follow-up rates between patients with and without NASH. The presence of NASH did not influence the follow-up rate of NAFLD patients, despite the fact that these patients were told that they were at increased risk of developing cirrhosis or HCC. This could be partly because NAFLD patients often do not have accurate and detailed knowledge on the natural course of NASH and the potential occurrence of HCC. The increased awareness of the risk of HCC in patients with chronic viral hepatitis has been established in Japan and could have increased the number of patients under surveillance. However, the present study indicates that patients in Japan are insufficiently aware that they are at increased risk of NASH progressing to cirrhosis or developing HCC. Also, we observed the similar percentage of patients who had been administered medication between patients who continued and discontinued regular follow-up visit. Therefore, the administration of medication did not prevent the drop out from regular visit in NAFLD patients. The medication that had been administered for NAFLD patients included ursodeoxycolic acid, or drugs for diabetes, hypertension, or hyperlipidemia, and was not drugs for NAFLD in the absence of definitive drugs for NAFLD. The emergence of drugs for NASH or NAFLD may increase the follow-up rate of patients with NAFLD in the future.

There are several limitations on this study. This is a retrospective study on the basis of the reviews of medical record. In addition, this is a study conducted in one region of Japan. All people in Japan are usually covered by a national medical insurance system, and the follow-up rates of patients with chronic hepatitis virus infection are very high. Therefore, the results observed in this study will not be applicable in other countries with a different medical insurance system or with different prevalence of patients with NAFLD and those with viral hepatitis.

## Conclusions

NAFLD patients had a low post-liver biopsy follow-up rate. Thus, it is important to continue the current efforts to inform individuals in Japan of the risks of this disease. Furthermore, given the large number of NAFLD patients, it will be important to further examine methods to identify patients with NAFLD who are at higher risk for disease progression or are at risk for developing HCC [14,15]. For patients chronically infected with HBV or HCV, chronic hepatitis virus infection is a risk factor for progression to cirrhosis or development of HCC. In contrast, it is not easy to identify NAFLD patients who are at high risk for progressing to cirrhosis or developing HCC. It will be necessary to further identify patients who are at increased risk for progressing to cirrhosis or developing HCC, even among patients who are pathologically confirmed to have NASH, in order to further clarify and better monitor patients with NASH who are at higher risk for disease progression and HCC, identifying patients who we should be strongly advised to have regular follow-up exams.

## List of abbreviations used

HBV: hepatitis B virus; HCC: hepatocellular carcinoma; HCV: hepatitis C virus; NAFLD: non-alcoholic fatty liver diseases; NASH: non-alcoholic steatohepatitis.

## Competing interests

The authors declare that they have no competing interests.

## Authors' contributions

All authors (HT, TK, SK, MT, YH, AK, and TT) are managed patients with viral hepatitis and those with NAFLD at an outpatient clinic. HT and TT performed liver biopsy. HT carried out the acquisition of patient data on follow-up status and performed statistical analyses. HT and TK participated the design of the study. HT drafted the manuscript. All authors read and approved the final manuscript.
